# Additional prognostic value of the BCT score in ER+HER2- breast cancer patients receiving a 21-gene assay-guided adjuvant treatments

**DOI:** 10.3389/fonc.2025.1517073

**Published:** 2025-04-16

**Authors:** Sung Gwe Ahn, Jonghan Yu, Seung Ho Baek, Yeon Jin Kim, Woo Young Kim, Jai Hyun Chung, Soong June Bae, Seok Won Kim, Seok Jin Nam, Gyungyub Gong, Young-Won Lee, Jai Hong Han, Joon Jeong, Sang Uk Woo, Eun-Gyeong Lee, Sae Byul Lee

**Affiliations:** ^1^ Department of Surgery, Gangnam Severance Hospital, Yonsei University College of Medicine, Seoul, Republic of Korea; ^2^ Institute for Breast Cancer Precision Medicine, Yonsei University College of Medicine, Seoul, Republic of Korea; ^3^ Breast Division, Department of Surgery, Samsung Medical Center, Sungkyunkwan University School of Medicine, Seoul, Republic of Korea; ^4^ Department of Breast and Endocrine Surgery, Korea University Guro Hospital, Seoul, Republic of Korea; ^5^ Department of Pathology, Asan Medical Center, University of Ulsan College of Medicine, Seoul, Republic of Korea; ^6^ Division of Breast and Endocrine Surgery, Department of Surgery, Korea University Anam Hospital, Korea University College of Medicine, Seoul, Republic of Korea; ^7^ Department of Surgery, Research Institute and Hospital, National Cancer Center, Goyang, Republic of Korea; ^8^ Department of Surgery, Asan Medical Center, University of Ulsan College of Medicine, Seoul, Republic of Korea

**Keywords:** breast neoplasm, estrogen receptor, endocrine therapy, 21-gene recurrence score, BCT score, prognostic factor

## Abstract

**Background:**

The 21-gene recurrence score (RS)-guided decision-making for adjuvant treatment has been utilized as a standard of care for early ER+HER2- breast cancer. We investigated a prognostic value of the Breast Cancer Test (BCT) score, a multigene assay incorporating clinical risk, in estrogen receptor (ER)+HER2- breast cancer patients receiving RS-guided adjuvant treatments, specifically focusing on chemotherapy-untreated patients with low RS.

**Methods:**

This multicenter cohort study included 759 patients who received RS-guided adjuvant treatment. The primary endpoint was recurrence-free survival (RFS), and the secondary endpoint was distant recurrence-free survival (DRFS).

**Results:**

At a median follow up of 85 months, the 7-year RFS was 92.9% (95% CI, 90.9%-94.9%). Among the 592 chemotherapy-untreated patients with low RS, the RFS differed significantly according to the BCT score (*P*=.014); the 7-year RFS was 95.5% (95% CI, 93.4%-97.7%) in the BCT-low group, while it was 89.9% (95% CI, 84.9%-95.1%) in the BCT-high group. The BCT score was an independent prognostic factor for both RFS and DRFS. In addition, the RFS of the low-BCT score group was superior to that of the high-BCT group in women aged 50 years or younger, with an RS of 16 to 25.

**Conclusions:**

Our study suggests the utility of the BCT score in stratifying the relapse risk among chemotherapy-untreated patients with a low RS, particularly in young women with an RS of 16–25 who are at risk for long-term recurrence.

## Introduction

In early estrogen receptor (ER)+human epidermal growth factor receptor-2 (HER2)- breast cancer, the use of a 21-gene expression assay to guide decision making for adjuvant treatments is widely accepted as part of the standard of care (1–4). This assay plays a crucial role in sparing chemotherapy for the majority of ER+HER2 − early breast cancer patients who exhibit a low genomic risk, as indicated by their 21-gene recurrence score (RS) (4, 5). Furthermore, it aids in accurately identifying those patients who could potentially benefit from chemotherapy (5, 6). However, it is noteworthy that chemotherapy could offer clinical benefits to premenopausal patients with a mid-range RS of 16-25 or those with a high clinical risk (3, 7).

Additionally, recent findings from the updated TAILOR-X trial, which included patients with node-negative, ER+HER2- breast cancer, have shown that the risk of late recurrence after 5 years surpasses that of early recurrence within the first 5 years (8). This line of evidence underscores the importance of gathering more real-world data on cohorts guided by 21-gene RS, particularly with longer follow-up periods.

The Breast Cancer Test (BCT) score is a multigene assay that incorporates clinical risk factors (9). Its primary purpose is to predict the risk of distant metastasis over a 10-year period in patients with hormone receptor (HR)+HER2- early breast cancer treated solely with anti-estrogen therapy (9, 10). The BCT score was computed based on the relative expression levels of six specific genes (*UBE2C, TOP2A, RRM2, FOXM1, MKI67*, and *BTN3A2*), as well as two clinical variables: nodal status and tumor size (9).

In our research, we aimed to investigate the additional value of the BCT score in a cohort of early stage, ER+HER2- breast cancer patients who were undergoing adjuvant treatments guided by a 21-gene expression assay. Specifically, our study focused on patients who did not receive chemotherapy and had low RS scores.

## Materials and methods

### Study population

This multi-center retrospective study adhered to the Strengthening the Reporting of Observational Studies in Epidemiology reporting guidelines for observational studies. We identified women with ER+HER2- invasive breast cancer who underwent curative surgery for ER+HER2- invasive breast cancer and received 21-gene RS-guided adjuvant treatment. Between March 2010 and April 2018, these patients received treatment at five academic institutions in South Korea: Gangnam Severance Hospital, Yonsei University College of Medicine, Samsung Medical Center, Sungkyunkwan University School of Medicine, National Cancer Center, Asan Medical Center, University of Ulsan College of Medicine, and Korea University Guro Hospital (11). The participating hospitals began to gradually implement the 21-gene RS test for patients with T1-2/N0-1, who consented to the test as part of their decision-making process for adjuvant treatments following surgery. Consequently, these patients received adjuvant endocrine treatments with or without chemotherapy, considering factors such as age, tumor stage, and personal preferences, all supervised by the RS. We collected clinical and pathological data for these patients by thoroughly reviewing their medical records, and the median age was 47 years. Data were collected between July and December 2022, encompassing the period from surgery to the last follow-up or death. This study adhered to the STROBE reporting guideline (12).

In a previous study (11), we acquired 871 Formalin-Fixed Paraffin-Embedded (FFPE) tissue samples from patients who had received 21-gene RS-guided adjuvant treatment at these five institutes. Among them, 771 patients were eligible for comparison between the GenesWell BCT score and 21-gene RS after excluding cases with FFPE tumor tissues that did not meet the GenesWell BCT test criteria or those with insufficient tumor or clinical information. In the current study, we included 759 patients after excluding patients with stage IV disease (n=1) or those with unknown survival status (n=11). A flowchart illustrating the patient selection process is shown in a consort diagram (**Supplementary Figure 1**).

Our study protocol was approved by the institutional review board of each participating institute. Given the retrospective nature of this study, the requirement for written informed consent was waived by the institutional review board.

### 21-gene recurrence score (Oncotype DX^®^) and BCT tests

For the 21-gene RS, we sent samples to Genomic Health for Oncotype DX testing prior to the study following established procedures as previously described (1, 4, 13, 14). To determine the BCT score, we extracted RNA from FFPE tissues and subjected samples with sufficient residual RNA for qRT-PCR, as described in previous studies (9). The BCT score was calculated using two clinical variables (tumor size and nodal status) in conjunction with the relative expression of nine genes: five proliferation genes (*UBE2C, TOP2A, RRM2, FOXM1, MKI67*), one immune gene (*BTN3A2*), and three reference genes (*CTBP1, CUL1, UBQLN1*). This scoring system was developed to estimate the prognosis of patients with ER+HER2- breast cancer. Following the initial disclosure of the previous study (9), case collection for the BCT score was conducted in a randomized manner, blinding the results of RS. The current study reports survival outcomes in a cohort with both RS and BCT scores.

### Categorization of risk groups

Patients were categorized into high-risk and low-risk BCT groups based on previously reported BCT scoring criteria (9). In summary, patients with a BCT score <4 were classified as low-risk, while those with a BCT score ≥4 were classified as high-risk. For the 21-gene RS test, patients were categorized into low-risk (RS <26) or high-risk (RS ≥26) groups using predefined cutoffs as established in the TAILOR-X and RxPONDER trials (3, 4, 6, 15). In an exploratory analysis of women aged 50 or younger, the low-risk group was further subdivided into low-range RS (0-15) and mid-range RS (16-25) (3). Thus, patients in this analysis were categorized into three groups: low-range RS (0-15), mid-range RS (16-25), and high RS (26-100). Clinical risk was determined using a modified version of the adjuvant! Online as in the MINDACT trial (1, 16).

### Statistical analysis

The primary endpoint of our study was recurrence-free survival (RFS), defined as the time interval between surgery and the occurrence of any tumor recurrence or mortality. The secondary endpoint was distant recurrence-free survival (DRFS), defined as the interval between surgery and the occurrence of distant metastasis or mortality. Partial likelihood ratio (LR) tests based on Cox proportional hazards regression models were used to test the prognostic information of RS and BCT score. We analyzed RFS and DRFS using Kaplan-Meier plots with log-rank tests and conducted multivariate Cox models, adjusting for conventional clinical variables, including T stage, N stage, and histologic grade. The pre-defined significance level was set at a two-sided alpha (α) <0.05. All statistical analyses using R 3.3.3 (http://r-project.org).

## Results

### Study population

This study included 759 patients. The baseline characteristics of the patients are summarized in **Supplementary Table 1**. The median age of the included patients was 47 years (range: 23-79). A total of 636 patients had invasive ductal carcinoma. Of these, 65.1% had T1 tumors, and 80.0% had node-negative disease. Adjuvant chemotherapy was administered to 19.7% of patients, while adjuvant radiotherapy was administered to 553 patients (72.9%). Progesterone receptor (PR) negativity was observed in 296 patients (12.9%), and grade 3 tumors were present in 225 patients (9.8%). Eighty-two patients (10.8%) received ovarian-function suppression (OFS) as a component of endocrine therapy. Regarding the level of clinical risk, 254 patients (42.9%) were classified as having high clinical risk.

Among these patients, 645 (85.0%) belonged to the low-RS group, and 520 (68.5%) had a low BCT score. We initially investigated the agreement between these two biomarkers (**Supplementary Figure 2**). Agreement was noted in 546 (71.9%) patients, whereas disagreement was observed in 213 (28.1%) patients. Among the patients with a low BCT score, 91.5% (476/520) had a low RS. However, among the patients with a high BCT score, 29.4% (70/239) had a high RS.

### Survival outcomes according to BCT score or RS

**Figure 1:**
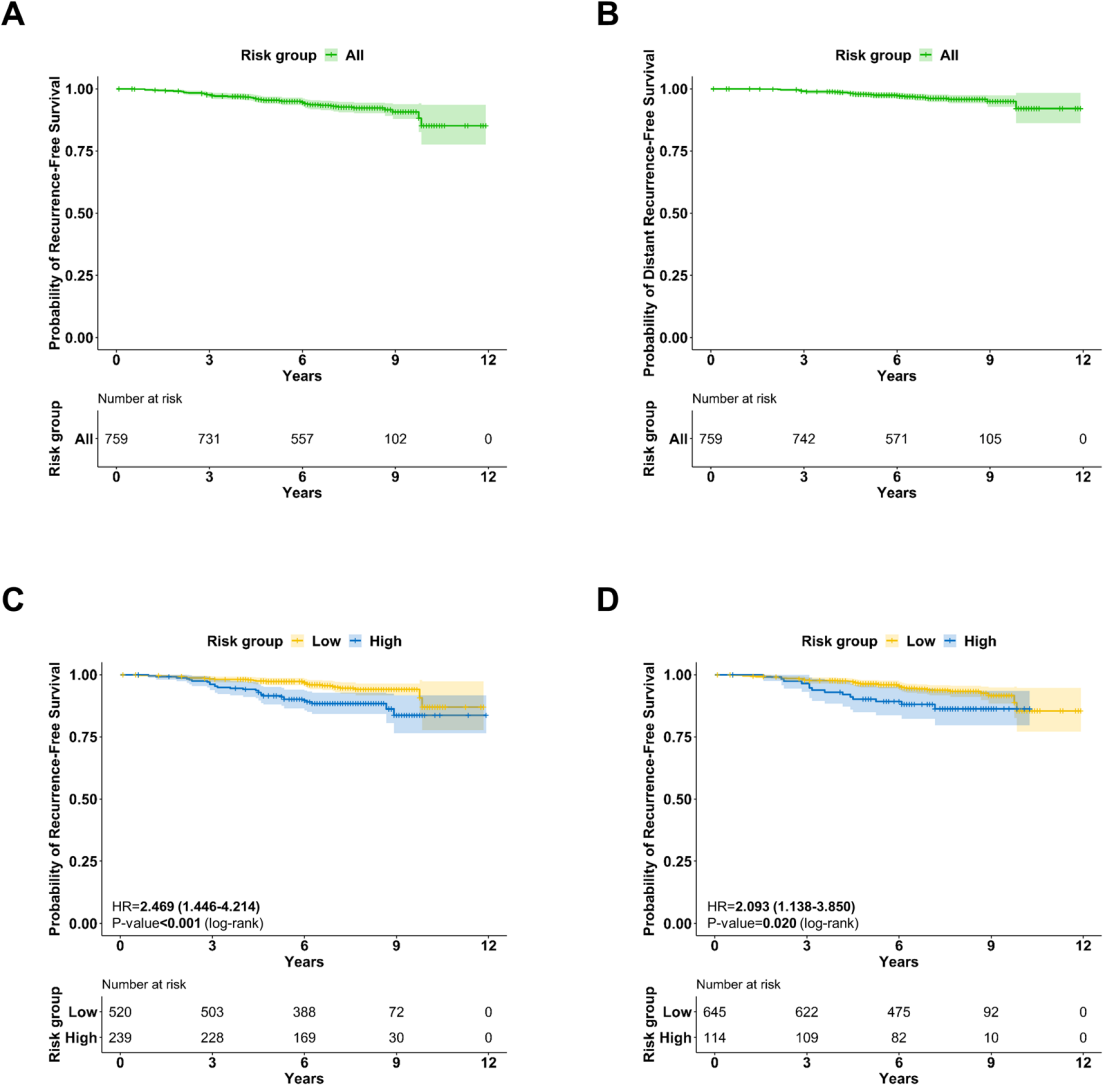
Survival outcomes **(A)** Recurrence-free survival for all patients **(B)** Distant recurrence-free survival for all patients **(C)** Recurrence-free survival by the BCT score (*P*<0.001, log-rank test) **(D)** Recurrence-free survival by the RS (*P*=.020, log-rank test).

At a median follow-up of 85 months, 54 patients had tumor recurrence and 28 had distant recurrence. The 7-year RFS and 7-year DRFS were 92.9% (95% CI, 90.9%-94.9%) and 96.2% (95% CI, 94.7%-97.7%), respectively (**Figures 1A, B**). When comparing RFS based on BCT score and RS, we noted significant differences. Specifically, the 7-year RFS was 95.0% (95% CI, 92.9%-97.1%) for the low BCT score group, in contrast to 88.4% (95% CI, 84.3%-92.7%) for the high BCT score group (**Figure 1C**; *P*<.001). Additionally, the 7-year RFS rates for the low-RS and high-RS groups were 93.7% (95% CI, 91.7%-95.8%) and 88.1% (95% CI, 82.2%-94.4%), respectively (**Figure 1D**; *P*=.020).

To assess the prognostic power of each multigene assay for RFS and DRFS, we performed likelihood-ratio tests (**Table 1**). Both RS and BCT score as either continuous or categorical value provided statistically significant prognostic information in predicting RFS and DRFS, while ΔLR χ2 of the continuous BCT score was highest among these.

**Table 1:** Likelihood-ratio tests for recurrence-free survival and distant recurrence-free survival in all patients.

	Univariable			Multivariable*	
		ΔLR χ2	*P*	ΔLR χ2	*P*
Recurrence-free survival	BCT score, *continuous*	21.822	<.0001	19.982	<.0001
	BCT score (<4 vs. ≥4), *categorial*	10.662	0.0011	8.916	0.0028
	RS	8.925	0.0028	3.839	0.0501
	RS (<26 vs. ≥26), *categorical*	4.899	0.0269	1.638	0.2006

	Univariable			Multivariable*	
		ΔLR χ2	*P*	ΔLR χ2	*P*
Distant recurrence-free survival	BCT score, *continuous*	14.297	0.0002	17.661	<.0001
	BCT score (<4 vs. ≥4), *categorial*	4.304	0.038	5.163	0.0231
	RS	7.634	0.0057	5.087	0.0241
	RS (<26 vs. ≥26), *categorical*	5.663	0.0173	3.655	0.0559

*Likelihood ratio test based on Cox proportional hazard models for univariate and multivariable analyses. Multivariable analyses were performed with age, tumor size, nodal status, grade, and progesterone receptor status. BCT, Breast Cancer Treatment; RS, Recurrence score.

### Prognostic value of BCT score in the group with low RS

**Figure 2:**
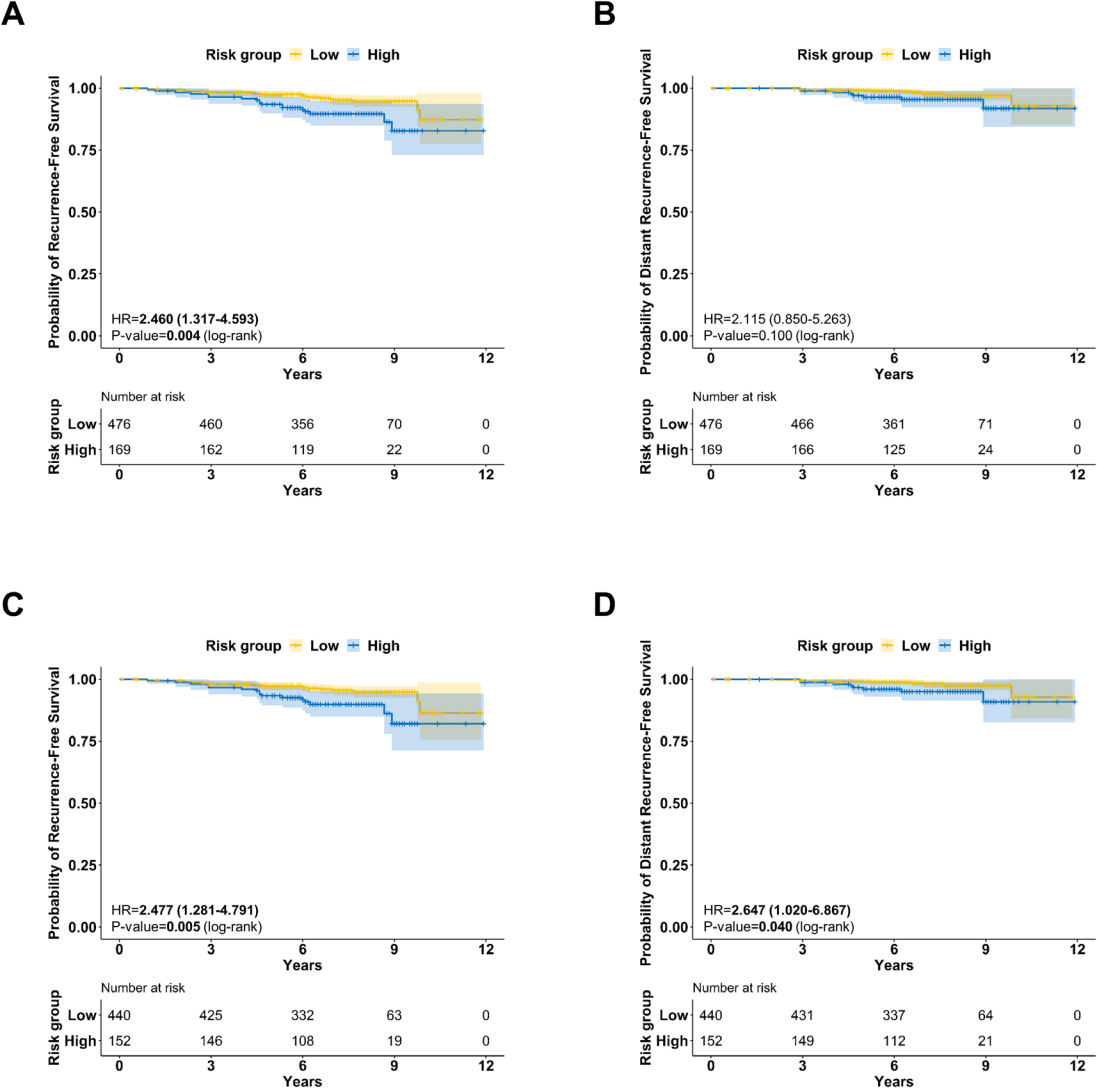
Survival analysis in patients with low RS (*N*=645): **(A)** Kaplan-Meier survival curve for RFS according to the BCT score (*P*=.004, log-rank test). **(B)** Kaplan-Meier survival curve for DRFS according to the BCT score (*P*=.010, log-rank test). Survival analysis of patients with low RS who did not receive chemotherapy (*N*=592). **(C)** Kaplan-Meier survival curve for RFS according to BCT score (*P*=.004, log-rank test). **(D)** Kaplan-Meier survival curve for DRFS according to BCT score (*P*=.040, log-rank test).

We then assessed the prognostic value of the BCT score in the two groups stratified by the RS. A high BCT score was significantly associated with an inferior RFS in the low RS group (n = 645). Specifically, the 7-year RFS was 95.2% (95% CI, 93.1%-97.4%) in the low BCT score group, compared to 89.6% (95% CI, 84.9%-94.6%) in the high BCT score group (**Figure 2A**; *P*=.004). Additionally, we observed that the 7-year DRFS for the low-BCT group was numerically higher than that for the high-BCT group, with rates of 97.5% (95% CI, 95.9%-99.2%) and 95.5% (95% CI, 92.2%-98.9%), respectively (**Figure 2B**; *P*=.10).

Within the low-RS group, 8.2% (53/645) of the patients underwent chemotherapy based on other clinical and pathological risk factors. We selected patients with low RS who did not receive chemotherapy and summarized their clinicopathological features and treatment modalities in **Table 2** (n=592). Of these patients, 440 (74.3%) had a low BCT score and 152 (25.7%) had a high BCT score.

**Table 2:** Clinical and pathological characteristics according to BCT score in chemotherapy-untreated group with low RS.

	Total (N=592)	Low BCT (%) (N=440, 74.3%)	High BCT (%) (N=152, 25.7%)	*P*-value
Age (years), median (range)	47 (23-79)	47 (23-79)	46 (28-75)	.516
Age Distribution				.540
≤ 50	399 (67.4)	293 (66.6)	106 (69.7)	
> 50	193 (32.6)	147 (33.4)	46 (30.3)	
Histologic type				.071
Ductal	488 (82.4)	372 (84.5)	116 (76.3)	
Lobular	58 (9.8)	38 (8.6)	20 (13.2)	
Other type or Mixed type	46 (7.8)	30 (6.8)	16 (10.5)	
T stage				<.001
T1	400 (67.6)	345 (78.4)	55 (36.2)	
T2	190 (32.1)	95 (21.6)	95 (62.5)	
T3	2 (0.3)	0 (0)	2 (1.3)	
N stage				<.001
N0	471 (79.6)	379 (86.1)	92 (60.5)	
N1	121 (20.4)	61 (13.9)	60 (39.5)	
Histologic Grade				.151
1 and 2	554 (93.6)	416 (94.5)	138 (90.8)	
3	38 (6.4)	24 (5.5)	14 (9.2)	
Clinical Risk (%)				<.001
High	254 (42.9)	133 (30.2)	121 (79.6)	
Low	338 (57.1)	307 (69.8)	31 (20.4)	
Progesterone receptor status				.629
Negative	38 (6.4)	30 (6.8)	8 (5.3)	
Positive	554 (93.6)	410 (93.2)	144 (94.7)	
Type of endocrine treatments				.559
Tamoxifen	379 (64.0)	287 (65.2)	92 (60.5)	
Aromatase Inhibitors	209 (35.3)	150 (34.1)	59 (38.8)	
Unknown	4 (0.7)	3 (0.7)	1 (0.7)	
Ovarian-function suppression				.025
Yes	81 (13.7)	52 (11.8)	29 (19.1)	
No	511 (86.3)	388 (88.2)	123 (80.9)	
Surgery				<.001
Breast-conservative surgery	428 (72.3)	337 (76.6)	91 (59.9)	
Mastectomy	164 (27.7)	103 (23.4)	61 (40.1)	
Adjuvant Radiotherapy				<.001
Yes	426 (72.0)	337 (76.6)	89 (58.6)	
No	166 (28.0)	103 (23.4)	63 (41.4)	

When we compared the clinical characteristics based on the BCT score, it became apparent that the high-BCT group had larger tumors, a higher prevalence of node-positive disease, more receipt of OFS, and a higher frequency of mastectomy (**Table 2**). Consequently, those with high BCT scores exhibited a higher frequency of high clinical risk. However, there were no discernible differences in age, histology, grade, or PR status between the two groups.

In this subset of patients with a low RS who did not receive chemotherapy, we noted significant differences in RFS and DRFS based on the BCT score. The 7-year RFS for the low-BCT group was higher than that for the high-BCT group, with rates of 95.5% (95% CI, 93.4%-97.7%) and 89.9% (95% CI, 84.9%-95.1%), respectively (**Figure 2C**; *P*=.004). Similarly, the 7-year DRFS was 98.0% (95% CI, 96.6%-99.5%) in the low BCT score group and 95.0% (95% CI, 91.4%-98.7%) in the high BCT score group (**Figure 2D**; *P*=.040). However, when we compared RFS and DRFS according to clinical risk, no significant survival differences were observed (**Supplementary Figure 3**).

We further explored the prognostic value of BCT score adjusted for conventional clinical variables within this subgroup. In the multivariate analysis for RFS, a high BCT score was identified as a significant risk factor for reduced RFS (**Table 3**; hazard ratio [HR]=3.175; 95% confidence interval [CI], 1.483–6.797; *P*=.003). Furthermore, in the multivariable analysis adjusted for T stage, nodal status, and grade, the BCT score emerged as an independent prognostic factor for DRFS (**Table 4**; HR=4.067; 95% CI, 1.397-11.834; *P*=.010). Additionally, in both multivariable models for RFS and DRFS, the continuous BCT score was found to be a significant prognostic factor (**Tables 3**, **4**).

**Table 3:** Multivariable analysis for RFS within low-RS and chemotherapy untreated group (*N*=592).

	Univariable			Multivariable		
	Hazard Ratio	95% Confidence Interval	*P*	Hazard Ratio	95% Confidence Interval	*P*
BCT score (<4 vs. ≥4), *categorical*	2.477	1.281-4.791	.007	3.175	1.483-6.797	.003
Size (≤2cm vs. >2cm)	0.996	0.489-2.031	.992	0.595	0.268-1.318	.201
Nodal status (Negative vs. Positive)	1.048	0.477-2.299	.908	0.766	0.336-1.748	.526
Grade (1/2 vs. 3)	2.370	0.921-6.099	.074	2.180	0.844-5.629	.107

	Univariable			Multivariable		
	Hazard Ratio	95% Confidence Interval	*P*	Hazard Ratio	95% Confidence Interval	*P*
BCT score (0 - 10), *continuous*	1.521	1.171-1.976	.002	1.723	1.245-2.384	.001
Size (≤2cm vs. >2cm)	0.996	0.489-2.031	.992	0.541	0.241-1.212	.135
Nodal status (Negative vs. Positive)	1.048	0.477-2.299	.908	0.724	0.316-1.659	.445
Grade (1/2 vs. 3)	2.370	0.921-6.099	.074	1.886	0.729-4.884	.191

BCT, Breast Cancer Treatment; RFS, Recurrence-free survival.

**Table 4:** Multivariable analysis for DRFS within low-RS and chemotherapy untreated group (*N*=592).

	Univariable			Multivariable		
	Hazard Ratio	95% Confidence Interval	*P*	Hazard Ratio	95% Confidence Interval	*P*
BCT score (<4 vs. ≥4), *categorical*	2.647	1.020-6.867	.045	4.067	1.397-11.834	.010
Size (≤2cm vs. >2cm)	0.688	0.224-2.114	.514	0.381	0.113-1.287	.120
Nodal status (Negative vs. Positive)	1.099	0.358-3.375	.869	0.712	0.220-2.305	.570
Grade (1/2 vs. 3)	0.893	0.118-6.735	.912	0.830	0.109-6.298	.857

	Univariable			Multivariable		
	Hazard Ratio	95% Confidence Interval	*P*	Hazard Ratio	95% Confidence Interval	*P*
BCT score (0 - 10), *continuous*	1.541	1.058-2.245	.002	1.947	1.210-3.133	.006
Size (≤2cm vs. >2cm)	0.688	0.224-2.114	.514	0.322	0.091-1.137	.078
Nodal status (Negative vs. Positive)	1.099	0.358-3.375	.869	0.648	0.197-2.130	.475
Grade (1/2 vs. 3)	0.893	0.118-6.735	.912	0.690	0.091-5.246	.720

BCT, Breast Cancer Treatment; DRFS, Distant recurrence-free survival.

### BCT score in subgroup of women aged 50 or younger with mid-range RS

Since the results of the intermediate RS group of the TAILOR-X trial were announced in 2018, the clinical benefit of chemotherapy was noted in the mid-range RS (16-25) for women aged ≤ 50 years (3). The agreement between the three RS categories and the binary BCT is provided in **Supplementary Table 2**. We evaluated the prognostic value of BCT within the subgroup of women aged ≤ 50 years with mid-range RS (16-25).

**Figure 3:**
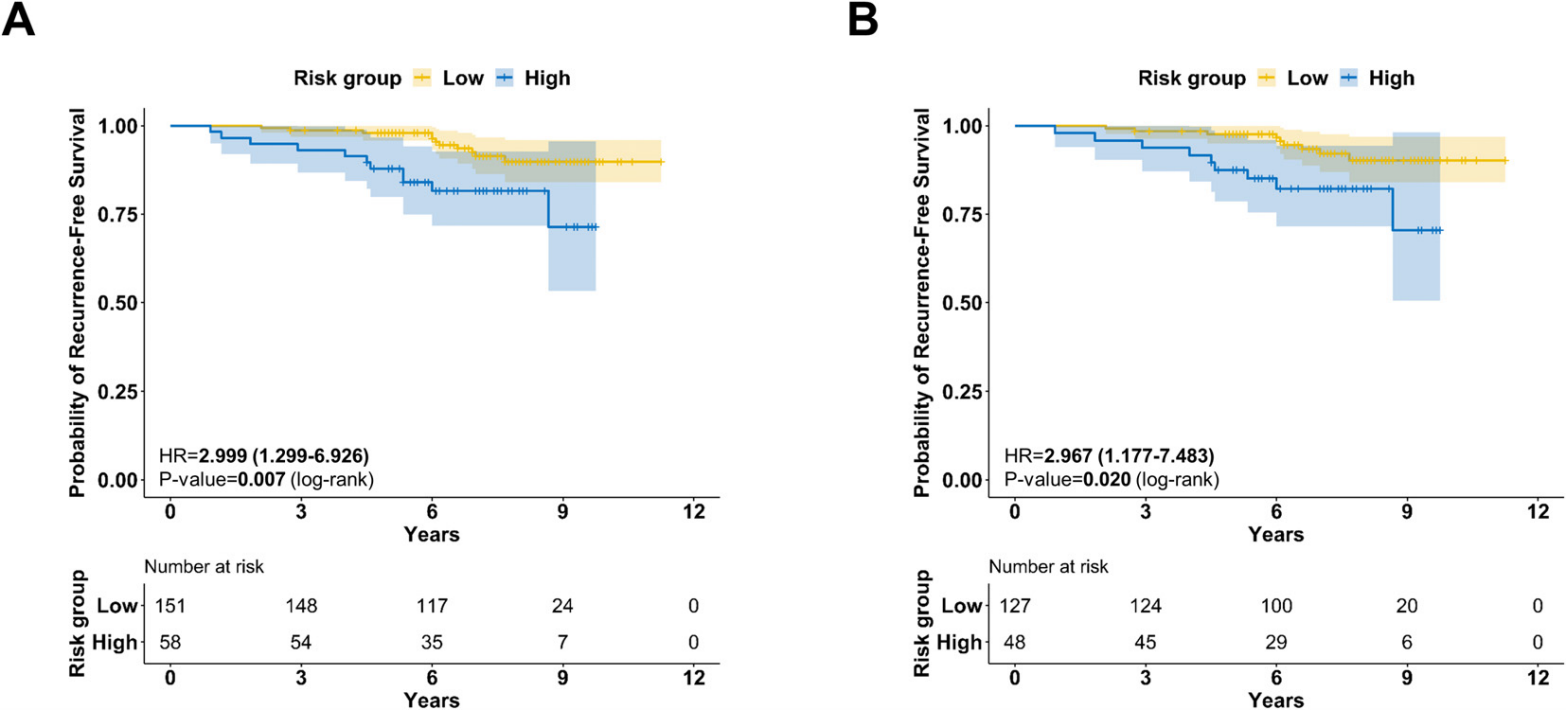
Survival analysis in the subgroup of patients aged 50 or younger with mid-range RS of 16-25 **(A)** All patients (N=209); Kaplan-Meier survival curve for RFS according to the BCT score (*P*=.007, log-rank test). **(B)** Chemotherapy-untreated patients (N=175); Kaplan-Meier survival curve for RFS according to the BCT score (*P*=0.020, log-rank test).

In our study, 209 patients were women aged 50 years or younger and had a mid-range RS (16-25). In this group, we observed that the RFS was significantly higher in the low BCT group (n=151) than in the high BCT group (n=58) (**Figure 3A**; *P*=.007). When excluding patients treated with chemotherapy (n=34), the RFS of the low BCT group remained superior to that of the high BCT group (**Figure 3B**; *P*=.020).

## Discussion

In our multicenter cohort of patients with early ER+HER2*-* breast cancer treated with RS-guided adjuvant treatment, we found that the BCT score has prognostic value. Specifically, within the low RS group that did not receive chemotherapy, the BCT score emerged as an effective tool for identifying patients at a higher risk of tumor recurrence or distant relapse. This added prognostic value of the BCT score is particularly prominent in women aged 50 years or younger, with a mid-range RS of 16-25. Our findings suggest that the BCT score can offer additional prognostic information beyond RS for early ER+HER2*-* breast cancer.

Overall, considering our cohort, which included 114 women (15.0%) with high RS (>26) or node-positive disease (20.0%), we observed favorable outcomes, with estimated 7-year RFS and 7-year DRFS rates of 92.9% and 96.2%, respectively. However, it is noteworthy that in the low-RS and chemotherapy-untreated groups, clinical stratification by BCT score resulted in a noticeable separation of survival curves. This divergence in survival curves began to manifest three years after surgery and became more pronounced around the five-year mark, indicating the potential for late recurrences in this group. Indeed, the 3-year RFS rates for the low- and high-BCT groups were 98.2% (95% CI, 96.9%-99.4%) and 96.7% (95% CI, 93.4%-99.6%), respectively, while the 5-year RFS rates were 97.2% (95.7%-98.8%) and 93.3% (89.4%-97.4%), respectively.

In another study using the BCT score, we reported that patients with a high BCT score had an increased risk of late relapse in a cohort with long-term follow-up, with a median follow-up of 17.4 years (17). Late recurrence is strongly associated with tumor size, nodal status (TN) of the primary tumor, and tumor grade (18–21). In this context, based on the TAILOR-X and B-14 trials, researchers developed a clinical tool that incorporates RS, tumor size, grade, and age (22).

In our study, late separation in survival based on the BCT score within the low-RS and chemotherapy-untreated groups was reasonable because this assay incorporates information related to TN. The BCT-high group had larger tumors, a higher prevalence of node-positive disease, and a higher clinical risk (**Table 1**). While the relatively higher tumor burden in the BCT-high group may partly contribute to increased recurrence, it cannot solely explain the inferior RFS, as the clinical risk stratification failed to differentiate survival curves in this group (**Supplementary Figure 3**).

Additionally, whether chemotherapy can reduce late recurrence in patients with ER+ breast cancer who have a relatively high tumor burden remains a subject of curiosity. RS was initially developed to identify biological properties that predict chemotherapy sensitivity in patients with ER+ breast cancer (5, 23). Therefore, the current practice of administering chemotherapy to individuals with high RS and omitting it for those with low RS has been established based on robust retrospective data and prospective trials (3–5, 7, 23). Interestingly, the recent updated findings of the TAILOR-X trial indicated that chemotherapy provided significant benefits in reducing distant recurrence among patients aged 50 years or younger with an RS of 16-20 and high clinical risk or an RS of 21-25 (8). More efforts are warranted to address late distant recurrence in premenopausal women with intermediate genomic risk, considering the TN of the primary tumor.

In contrast to several other multigene assays for determining the necessity of chemotherapy in ER+ breast cancer, the BCT score does not include ER-related genes (9). Unlike the BCT score, the vast majority of multigene assays, including the 21-gene expression assay and Mammaprint^®^, incorporate ER modules (5, 16, 23, 24). Consequently, ER and PR expression can affect the final score of the assay. This may explain why the BCT score is not as influenced by the estrogen-enriched hormonal milieu often present in young women, which can lead to increased gene expression related to the ER modules. In fact, in patients aged ≤ 50 years with an RS of 16-25, the BCT score provided prognostic information regardless of chemotherapy receipt (**Figure 3**). Further data are needed to ascertain the role of BCT score in guiding adjuvant treatment for premenopausal women.

Our study had several limitations. Our patients received RS-guided adjuvant treatment before the primary release of randomized controlled trial data, especially those with intermediate RS (11-25) in the TAILOR-X trial in 2018 (3). Therefore, treatment decision-making may differ from the current guidelines. Additionally, the RS cutoff varies based on patient age, menopausal status, and level of clinical risk (3, 7). Moreover, when patients receive results indicating both a low RS and high BCT score, our findings do not provide clear guidance regarding chemotherapy. Lastly, given the late recurrence observed in the updated 12-year data from TAILOR-X (8), longer follow-up is required. Nonetheless, our findings highlight the potential of the BCT score to provide prognostic information, especially for patients in the grey zone and young individuals.

In summary, we found that the BCT score was valuable for stratifying the risk of relapse in chemotherapy-untreated patients with a low RS. This indicates that the BCT score could provide additional clinical value in identifying patients with a long-term risk of relapse, particularly in young women with an RS of 16–25. Our findings imply that the BCT score may help further personalized treatment for these patients.

## Data Availability

The original contributions presented in the study are included in the article/**Supplementary Material**. Further inquiries can be directed to the corresponding authors.

## References

[1] Bae SJ Ahn SG Ji JH Chu C Kim D Lee J. Application of the 21-gene recurrence score in patients with early HR-positive/HER2-negative breast cancer: chemotherapy and survival rate according to clinical risk. Cancers (Basel). (2021) 13(16):4003. doi: 10.3390/cancers13164003 34439158 PMC8394098

[2] Kwa M Makris A Esteva FJ. Clinical utility of gene-expression signatures in early stage breast cancer. Nat Rev Clin Oncol. (2017) 14:595–610. doi: 10.1038/nrclinonc.2017.74 28561071

[3] Sparano JA Gray RJ Makower DF Pritchard KI Albain KS Hayes DF. Adjuvant chemotherapy guided by a 21-gene expression assay in breast cancer. N Engl J Med. (2018) 379:111–21. doi: 10.1056/NEJMoa1804710 PMC6172658 29860917

[4] Sparano JA Gray RJ Makower DF Pritchard KI Albain KS Hayes DF. Prospective validation of a 21-gene expression assay in breast cancer. N Engl J Med. (2015) 373:2005–14. doi: 10.1056/NEJMoa1510764 PMC4701034 26412349

[5] Paik S Tang G Shak S Kim C Baker J Kim W. Gene expression and benefit of chemotherapy in women with node-negative, estrogen receptor-positive breast cancer. J Clin Oncol. (2006) 24:3726–34. doi: 10.1200/jco.2005.04.7985 16720680

[6] Sparano JA Gray RJ Makower DF Albain KS Saphner TJ Badve SS. Clinical outcomes in early breast cancer with a high 21-gene recurrence score of 26 to 100 assigned to adjuvant chemotherapy plus endocrine therapy: A secondary analysis of the TAILORx randomized clinical trial. JAMA Oncol. (2020) 6:367–74. doi: 10.1001/jamaoncol.2019.4794 PMC6777230 31566680

[7] Sparano JA Gray RJ Ravdin PM Makower DF Pritchard KI Albain KS. Clinical and genomic risk to guide the use of adjuvant therapy for breast cancer. N Engl J Med. (2019) 380:2395–405. doi: 10.1056/NEJMoa1904819 PMC6709671 31157962

[8] Sparano J Gray RJ Makower D Albain KS Hayes DF Geyer C. Abstract GS1-05: trial assigning individualized options for treatment (TAILORx): an update including 12-year event rates. Cancer Res. (2023) 83. doi: 10.1158/1538-7445.Sabcs22-gs1-05

[9] Gong G Kwon MJ Han J Lee HJ Lee SK Lee JE. A new molecular prognostic score for predicting the risk of distant metastasis in patients with HR+/HER2- early breast cancer. Sci Rep. (2017) 7:45554. doi: 10.1038/srep45554 28350001 PMC5368569

[10] Kwon MJ Lee SB Han J Lee JE Lee JW Gong G. BCT score predicts chemotherapy benefit in Asian patients with hormone receptor-positive, HER2-negative, lymph node-negative breast cancer. PloS One. (2018) 13:e0207155. doi: 10.1371/journal.pone.0207155 30462685 PMC6248959

[11] Kwon MJ Lee JE Jeong J Woo SU Han J Kang BI. Comparison of genesWell BCT score with oncotype DX recurrence score for risk classification in asian women with hormone receptor-positive, HER2-negative early breast cancer. Front Oncol. (2019) 9:667. doi: 10.3389/fonc.2019.00667 31404265 PMC6670782

[12] von Elm E Altman DG Egger M Pocock SJ Gøtzsche PC Vandenbroucke JP. The Strengthening the Reporting of Observational Studies in Epidemiology (STROBE) statement: guidelines for reporting observational studies. J Clin Epidemiol. (2008) 61:344–9. doi: 10.1016/j.jclinepi.2007.11.008 18313558

[13] Lee J Kim H Bae SJ Ji JH Lee JW Son BH. Association of body mass index with 21-gene recurrence score among women with estrogen receptor-positive, ERBB2-negative breast cancer. JAMA Netw Open. (2022) 5:e2243935. doi: 10.1001/jamanetworkopen.2022.43935 36441548 PMC9706366

[14] Lee J Lee YJ Bae SJ Baek SH Kook Y Cha YJ. Ki-67, 21-gene recurrence score, endocrine resistance, and survival in patients with breast cancer. JAMA Netw Open. (2023) 6:e2330961. doi: 10.1001/jamanetworkopen.2023.30961 37647069 PMC10469325

[15] Kalinsky K Barlow WE Gralow JR Meric-Bernstam F Albain KS Hayes DF. 21-gene assay to inform chemotherapy benefit in node-positive breast cancer. N Engl J Med. (2021) 385:2336–47. doi: 10.1056/NEJMoa2108873 PMC9096864 34914339

[16] Cardoso F van’t Veer LJ Bogaerts J Slaets L Viale G Delaloge S. 70-gene signature as an aid to treatment decisions in early-stage breast cancer. N Engl J Med. (2016) 375:717–29. doi: 10.1056/NEJMoa1602253 27557300

[17] Fujiki Y Kashiwaba M Sato M Kawano J Teraoka M Kanemitsu S. Long-term prognostic value of the GenesWell BCT score in Asian women with hormone receptor-positive/HER2-negative early breast cancer. Breast Cancer. (2024) 31:31–41. doi: 10.1007/s12282-023-01509-7 37812303 PMC10764379

[18] Ahn SG Lee HM Cho SH Bae SJ Lee SA Hwang SH. The difference in prognostic factors between early recurrence and late recurrence in estrogen receptor-positive breast cancer: nodal stage differently impacts early and late recurrence. PloS One. (2013) 8:e63510. doi: 10.1371/journal.pone.0063510 23717438 PMC3661516

[19] Dowling RJO Kalinsky K Hayes DF Bidard FC Cescon DW Chandarlapaty S. Toronto workshop on late recurrence in estrogen receptor-positive breast cancer: part 1: late recurrence: current understanding, clinical considerations. JNCI Cancer Spectr. (2019) 3:pkz050. doi: 10.1093/jncics/pkz050 32337479 PMC7049988

[20] Kennecke HF Olivotto IA Speers C Norris B Chia SK Bryce C. Late risk of relapse and mortality among postmenopausal women with estrogen responsive early breast cancer after 5 years of tamoxifen. Ann Oncol. (2007) 18:45–51. doi: 10.1093/annonc/mdl334 17030545

[21] Pan H Gray R Braybrooke J Davies C Taylor C McGale P. 20-Year Risks of Breast-Cancer Recurrence after Stopping Endocrine Therapy at 5 Years. N Engl J Med. (2017) 377:1836–46. doi: 10.1056/NEJMoa1701830 PMC5734609 29117498

[22] Sparano JA Crager MR Tang G Gray RJ Stemmer SM Shak S. Development and validation of a tool integrating the 21-gene recurrence score and clinical-pathological features to individualize prognosis and prediction of chemotherapy benefit in early breast cancer. J Clin Oncol. (2021) 39:557–64. doi: 10.1200/jco.20.03007 PMC8078482 33306425

[23] Paik S Shak S Tang G Kim C Baker J Cronin M. A multigene assay to predict recurrence of tamoxifen-treated, node-negative breast cancer. N Engl J Med. (2004) 351:2817–26. doi: 10.1056/NEJMoa041588 15591335

[24] Piccart M van ‘t Veer LJ Poncet C Lopes Cardozo JMN Delaloge S Pierga JY. 70-gene signature as an aid for treatment decisions in early breast cancer: updated results of the phase 3 randomised MINDACT trial with an exploratory analysis by age. Lancet Oncol. (2021) 22:476–88. doi: 10.1016/s1470-2045(21)00007-3 33721561

