# Supplementary material for: Additional prognostic value of the BCT score in ER+HER2- breast cancer patients receiving a 21-gene assay-guided adjuvant treatments
Source: Front Oncol. 2025 Apr 16;15:1517073. doi: 10.3389/fonc.2025.1517073 (PMC12040951; doi:10.3389/fonc.2025.1517073)
Supplement: Supplementary file 1 [file DataSheet1.docx]

**Supplementary Figure 1.** Consort diagram of enrolled patients

RS, recurrence score


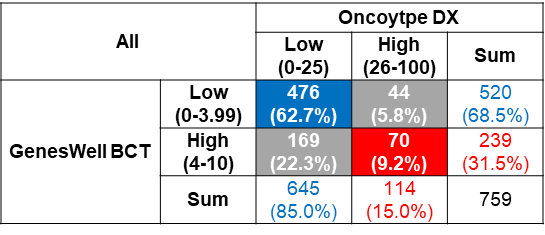


**Supplementary Figure 2.** **Association between BCT score and 21-gene RS**

The cutoffs for the BCT score and RS were set at 4 and 26, respectively. Of the patients, 645 (85.0%) belonged to the low-RS group, and 520 (68.5%) had a low BCT score. Agreement was observed in 546 (71.9%) patients, while disagreement was noted in 213 (28.1%). Among patients with a low BCT score, 91.5% (476/520) also had a low RS. However, among patients with a high BCT score, 29.4% (70/239) had a high RS.

**
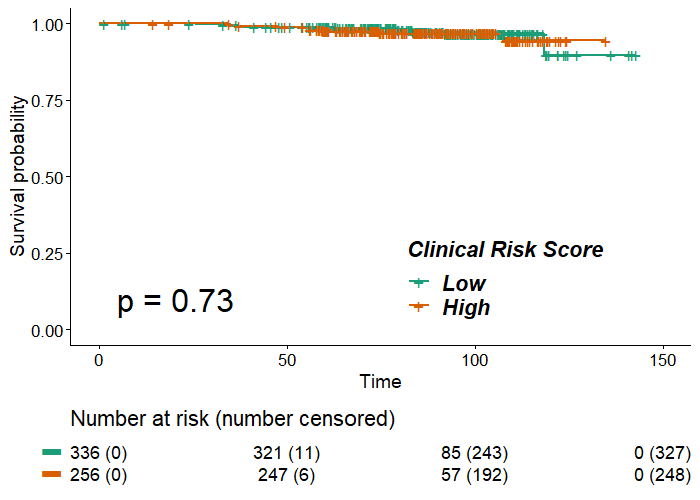
**

months

**(B) DRFS by Clinical Risk Score**

**Supplementaryary Figure 3.** Kaplan-Meier survival curve by the level of clinical risk. (A) Comparison of RFS according to the level of clinical risk (*P*=.11); (B) Comparison of DRFS according to the level of clinical risk (*P*=.73).

DRFS, distant recurrence-free survival; RS, recurrence score; RFS, recurrence-free survival.

**Supplementary Table 1. Baseline characteristics of enrolled patients**

|  | Enrolled patients (%) (N=759) |
| --- | --- |
| Age (years), median (range) | 47 (23-79) |
| Age Distribution (%) |  |
| ≤ 50 | 505 (66.5) |
| > 50 | 254 (33.5) |
| Histologic type (%) |  |
| Ductal | 636 (83.8) |
| Lobular | 65 (8.6) |
| Other type or Mixed type | 58 (7.6) |
| T stage (%) |  |
| T1 | 495 (65.1) |
| T2 | 261 (34.5) |
| T3 | 3 (0.4) |
| N stage (%) |  |
| N0 | 608 (80.0) |
| N1 | 151 (20.0) |
| Histologic Grade (%) |  |
| 1 and 2 | 668 (88.0) |
| 3 | 91 (12.0) |
| Clinical Risk |  |
| High | 360 (47.4) |
| Low | 399 (52.6) |
| Progesterone receptor status (%) |  |
| Negative | 76 (10.0) |
| Positive | 683 (90.0) |
| Surgery |  |
| Breast-conservative surgery | 555 (73.1) |
| Mastectomy | 204 (26.9) |
| Adjuvant Chemotherapy (%) |  |
| Yes | 149 (19.6) |
| No | 610 (80.4) |
| Type of endocrine treatments (%) |  |
| Tamoxifen | 478 (62.9) |
| Aromatase Inhibitor | 276 (36.3) |
| Unknown | 5 (0.8) |
| Ovarian-Function suppression (%) |  |
| Yes | 82 (10.8) |
| No | 678 (89.2) |
| Adjuvant Radiotherapy (%) |  |
| Yes | 553 (72.9) |
| No | 206 (27.1) |

**Supplementary Table 2. Agreement between three RS categories and the binary BCT score**

|  |  | **low-range RS:**  **0-15**  **(N=347)** | **mid-range RS:**  **16-25**  **(N=299)** | **High RS:**  **26-100**  **(N=114)** |
| --- | --- | --- | --- | --- |
| **BCT score** | < 4.0 (Low) | 261 (75.2) | 215 (71.9) | 44 (38.6) |
|  | ≥ 4.0 (High) | 86 (24.8) | 84 (28.1) | 70 (61.4) |
